# Supplementary material for: Combining rapid antigen testing and syndromic surveillance improves community-based COVID-19 detection in a low-income country
Source: Nat Commun. 2022 May 26;13:2877. doi: 10.1038/s41467-022-30640-w (PMC9135686; doi:10.1038/s41467-022-30640-w)
Supplement: Supplementary file 1 — Supplementary Information [file 41467_2022_30640_MOESM1_ESM.pdf]

Supplementary Materials for: Combining Rapid Antigen Testing and Syndromic Surveillance Improves Community-Based COVID-19 Detection in Low-and-Middle-Income Countries

Fergus J Chadwick^*[a,b], Jessica Clark[a,b], Shayan Chowdhury[c], Tasnuva Chowdhury[a], David J Pascall[d], Yacob Haddou[a,b], Joanna Andrecka[e], Mikolaj Kundegorski[f,b], Craig Wilkie[f,b], Eric Brum[e], Tahmina Shirin[g], A S M Alamgir[g], Mahbubur Rahman[g], Ahmed Nawsher Alam[g], Farzana Khan[g], Ben Swallow[f,b], Frances Mair[h], Janine Illian[f,b], Caroline L Trottter[i], Davina L Hill[a,b], Dirk Husmeier[f], Jason Matthiopoulos[a,b], Katie Hampson[a,b], Ayesha Sania[j]

[a] *Institute of Biodiversity Animal Health and Comparative Medicine University of Glasgow*

[b] *COVID-19 in LMICs Research Group University of Glasgow*

[c] *a2i United Nations Development Program ICT Ministry Bangladesh*

[d] *MRC Biostatistics Unit University of Cambridge*

[e] *Food and Agriculture Organisation of the United Nations in support of the UN Interagency Support Team Bangladesh*

[f] *School of Mathematics and Statistics University of Glasgow*

[g] *Institute of Epidemiology Disease Control and Research Ministry of Health Bangladesh*

[h] *General Practice and Primary Care Institute of Health and Wellbeing University of Glasgow*

[i] *Departments of Pathology and Veterinary Medicine University of Cambridge Cambridge United Kingdom*

[j] *Division of Developmental Neuroscience Department of Psychiatry Columbia University*

---

*Corresponding author
Email address: `fergusjchadwick@gmail.com` (Fergus J Chadwick^*)
[1]Corresponding Author

1. Supplementary Figures

The key result we would like to highlight in the paper is the power of the workflow we have developed. Individual parameter estimates will vary depending on the population to which the models are applied and we strongly advise against applying the parameter values found here (for example, the final symptoms chosen through model selection) outwith the population and time to which the models were fit.

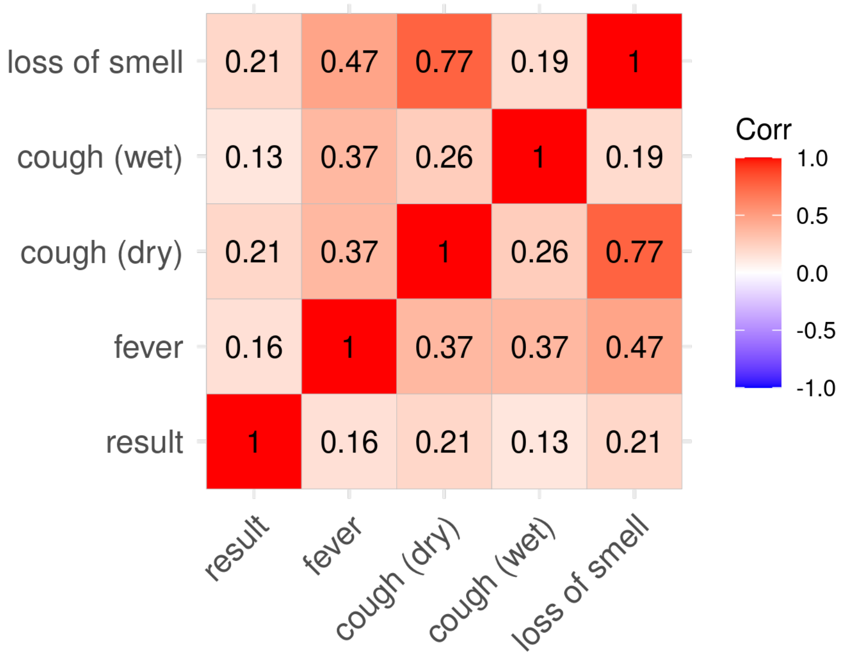

Supplementary Figure 1: Median correlation between PCR result and top symptoms for 4 symptom Syndromic-only Model

1.1. Correlation Estimates

The relationship between symptoms and results can only be understood through the full correlation matrix. In Figures 1 and 2, we present the median correlations for the four symptom Syndromic-only and Syndromic-RAT Combined models. These results should not be used to prioritise future data collection because the most predictive symptoms are liable to change with time (e.g., the emergence of new COVID variants) and context (e.g., broader vaccination levels).

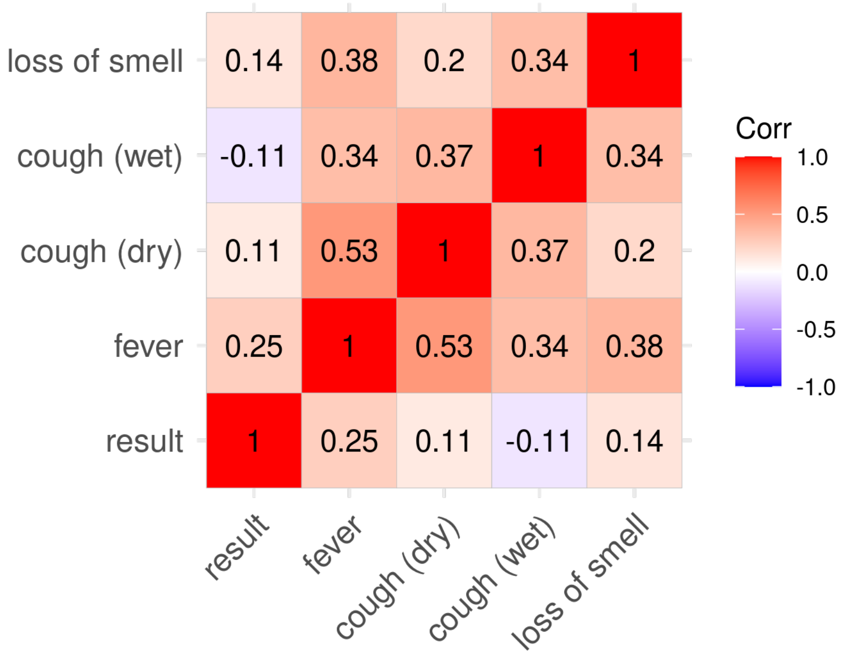

Supplementary Figure 2: Median correlation between PCR result and top symptoms for 4 symptom Syndromic-RAT Combined Model

Supplementary Table 1: Translation of best model performance by scenario into number of patients per 1000 tested who were incorrectly diagnosed, broken down by case positivity rate (CPR). CPRs chosen to reflect low, average and high values in Bangladesh.

		Per 1000					
		5% CPR		20% CPR		35% CPR	
Model Class	Scenario	False Positives	False Negatives	False Positives	False Negatives	False Positives	False Negatives
RATonly	All	24	20	21	81	17	141
SyndOnly	1	400	21	337	85	274	149
SyndRAT	1	105	18	88	71	72	124
SyndOnly	2	703	10	592	40	481	69
SyndRAT	2	392	10	330	39	268	68
SyndOnly	3	189	34	159	137	129	240
SyndRAT	3	188	15	158	59	129	103

2. Supplementary Tables

2.1. Translation of Error Rates into Raw Numbers Based on Case Positivity Rate

False positive and false negative rates can only be translated into numbers of people affected if the case positivity rate is known. To demonstrate how the numbers of misclassifications change for the same false positive and false negative rates, we have scaled these numbers with respect to low (5%), average (20%) and high (35%) CPRs in Bangladesh in Table 1.

3. Supplementary Methods

Below we have extended the modelling description provided in the main text to include more technical detail. The code used to implement these tasks is available at https://github.com/fergusjchadwick/COVID19_SyndromicRAT_public.

*3.1. **Modelling***

*3.1.1. **Structure***

We examined the ability of the two imperfect identification methods, syndromic modelling and rapid antigen testing (RAT), to predict the patient's COVID-19 status when used separately and together. These combinations define three model classes (Main Text Figure 4).

RAT-only uses only the RAT result. It equates being RAT-positive with the patient being PCR-positive for COVID-19 (hereafter, PCR-positive), and being RAT-negative with PCR-negativity.

Syndromic-only uses only the syndromic data. For this model, we used a Bayesian multivariate probit model.[1] The multivariate probit structures the outcomes of the PCR test and symptoms presence/absence as a D-dimensional vector of binary outcomes ($\boldsymbol{y}_i = (y_{i1}, y_{i2}, \ldots, y_{id})$, $y_{ij} \in \{0, 1\}$). These outcomes are determined by an indicator function which takes a D-dimensional vector of *continuous latent* variables ($\boldsymbol{z}_i = (z_{i1}, z_{i2}, \ldots, z_{iD})$, $z_{ij} \in \mathbb{R}$). These latent continuous variables then covary as realisations of a D-dimensional multivariate normal, with the mean of the error structure informed by a linear predictor (in our case formed of the covariates age and gender), $\sum_{j=1}^{J} x_{ij}\beta_{jd} + \epsilon_{id}$, and a covariance ($\Sigma$) between dimensions. The linear predictor allows us to condition the outcomes on risk factor variables (here, age and gender). The covariance structure allows us to account for the correlated nature of the symptoms with each other and the outcome. This multivariate approach (multiple response variables) is also a very efficient way of encoding complex relationships between symptoms. These relationships need to be accounted for because symptoms are not simply additive in their predictive power. For example, in the diagnosis of measles the "Three C's" are used: cough, coryza (irritation and inflammation of the mucous membrane in the nose leading to head cold, fever, sneezing) and conjunctivitis. These symptoms individually, and in pairwise combination could be indicative of a wide range of diseases, but when all three are present measles is a highly probable cause (obviously, this is a simplified example conditioning on patient age and vaccination status). In the alternative, univariate approach, symptoms would be encoded as covariates in the linear predictor for PCR-status, and the complex relationships would need to be reflected as high-order interaction terms. These interaction terms use a large number of parameters and can be hard to fit to

data. Using a multivariate structure allows us to exploit more efficient posterior sampling algorithms, and in higher dimensional settings like this uses fewer parameters.

The covariance matrix formulation of the model described above is not identifiable, because the variance, $diag(\Sigma)$ and means of the latent variables, $\boldsymbol{z}_i$ trade off against each other.[1] For this reason, we use a correlation matrix, Ω, formulation with the variance set to 1. A correlation based framework also makes communication with clinicians and other practitioners smoother as correlations are more familiar. We thus formulate the multivariate probit as:

$$
\begin{aligned}
y_{id} &= \mathbb{I}(z_{id} > 0) \\
\boldsymbol{z}_i &= \boldsymbol{x}_i \boldsymbol{\beta} + \boldsymbol{\epsilon}_i \\
z_{id} &= \sum_{j=1}^{J} x_{ij}\beta_{jd} + \epsilon_{id} \\
\boldsymbol{\epsilon}_i &\sim N(\mathbf{0}, \boldsymbol{\Omega}) \\
\Omega_{ii} &= 1 \\
\beta &\sim N(0, 1) \\
\boldsymbol{\Omega} &\sim \text{LKJ}(1)
\end{aligned}
\tag{1}
$$

Syndromic-RAT Combined combines the two data sources. We utilise the specificity of RAT by treating RAT-positive patients as PCR-positive patients. The RAT-negative patients are modelled using the sensitive syndromic approach using Syndromic-only to capture PCR-positive patients that are missed by the RAT. This approach leverages the potential different syndromic profiles of PCR-positve patients who are RAT-positive and -negative, allowing the model to adapt solely to the latter. Structurally, the model combines RAT-only and Syndromic-only, with RAT-positive patients being modelled using RAT-only, and RAT-negative patients with Syndromic-only.

By using a Bayesian formulation, we generate full posteriors for our parameter estimates, allowing natural quantification of uncertainty. Bayesian methods also facilitate the use of more informative priors. We used minimally informative priors here. For covariate coefficients (betas) we used standard normals which are relatively flat in the probit scale. For the correlation prior, we used the Lewandowski-Kurowicka-Joe (LKJ) distribution, a covariance matrix prior with unit variance (i.e. a prior for correlation matrices). The LKJ distribution has a single parameter, η, which controls the degree of marginal correlation shrinkage. We used minimal shrinkage, $\eta = 1$)[4]. More informative priors that incorporate spatio-temporal effects, for instance, would be natural extensions. The models were fitted to the data using Bayesian inference techniques based on Hamiltonian Monte Carlo in the Stan programming language[2]. The models all converged with zero divergent transitions and large effective sample

sizes.

3.1.2. Model Selection

We conducted backwards model selection (starting with the most complex, biologically plausible model) to identify a subset of models with the highest predictive power under temporal cross-validation (Main Text Figure 5). For the cross-validation, we divided the data into 5 folds of equal sizes in time order (i.e. the first fold is formed of the chronologically first $\frac{N}{K}$ patients, where N is the number of patients and K is the number of folds, the second fold by the next $\frac{N}{K}$ etc.) To test the sensitivity of this cross-validation structure, we also did a strict temporal division (i.e. the first $\frac{T}{K}$ days where T is the number of days samples were taken on). The results did not change qualitatively between these approaches.

The coarse round of model selection (Main Text Figure 5) selected candidate symptoms based on whether they had a strong and consistent correlation with PCR as estimated according to Equation (1). The models were fit with both covariates throughout the coarse round and symptoms were compared in nested models. In the fine round of model selection, these candidate symptoms and the covariate combinations (age and gender, age, gender and no covariates) were permuted to more exhaustively explore the model space. Reducing the number of possible models using the two stages of model selection was necessary to reduce computational demand and reduce the risk of overfitting models to the test scenarios. The large number of symptoms corresponds to a high number of potential model configurations (>131 000 for 14 symptoms and two covariates) which might perform well on the test sets (even under the challenging conditions of temporal cross-validation) but lack transferability.

By using general predictive power to narrow down the number of candidate models and then testing those models, we are more likely to choose models that generalise well to new data. It was clear when fitting the models that there were “jumps” in performance (as defined below) between models containing five and four symptoms, so the models with one to four symptoms were used as the candidate models. Zero symptom models were not included in the analysis as they do not correspond to a feasible policy (with covariates they would require governments to ask individuals of a given gender and age as COVID-19 positive, and without covariates they would involve randomly assigning individuals as COVID-19 positive).

3.1.3. Predictive Performance

We scored the models’ predictive power using binary cross-entropy (hereafter, cross-entropy). Cross-entropy measures the accuracy of models that generate probabilities of binary outcomes, rather than make binary classifications,

similar in concept to a mean square error for normally-distributed data, but adapted for binary data.[3] A cross-entropy value close to zero corresponds to high levels of accuracy, with larger values indicating lower accuracy. More specifically, the metric allows us to compare a binary vector, $\boldsymbol{y} \in [0, 1]$, with a vector of probabilistic predictions ($p(\boldsymbol{y}) \in (0, 1)$) as follows:

$$\boldsymbol{H}_p(q) = -\frac{1}{N}\sum_{i=1}^{N} y_i \cdot log(p(y_i)) + (1 - y_i) \cdot log(1 - p(y_i)) \tag{2}$$

The resulting score is comparable across all methods for assigning predictions where the same test data are used, allowing us to compare predictions from Model Classes 1-3. $H_p(q) \in 0, \boldsymbol{R}_+$ with zero indicating perfect prediction (assigning probabilities of ones and zeroes to outcomes of ones and zeros exactly) and larger values indicating worse predictions.

3.1.4. Classification Performance

In applied settings, models must often be evaluated on their performance as classifiers rather than just as prediction engines (i.e. their ability to say a patient is COVID-19 positive or negative, not simply the probability the patient might be COVID-19 positive or negative). To generate a classification, $\hat{Y}$, a probability threshold, $\hat{p}$, must be chosen over which patients are classified as COVID-19 positive:

$$\hat{Y} = \begin{cases} 1, & \text{if } p(y) \geq \hat{p} \\ 0 & \text{otherwise} \end{cases} \tag{3}$$

Receiver operating characteristics (ROCs) are a way to measure the performance of a set of classifications in terms of true and false positives and negatives (TP, FP, TN and FN) and the rates of each of these classification types (e.g. $TPR = \frac{TP}{TP+FN}$, and $FPR = \frac{FP}{FP+TN}$). The error rates are calculated with respect to a particular threshold, $\hat{p}$, or across the range of possible $\hat{p}$s to generate a ROC curve. In our epidemiological scenarios (outlined below) we use our ROC curve calculations to identify single thresholds which yield a required error rate.

We strongly emphasise that generic performance here is only used to show the flexibility of the model classes; the best model for a local situation can only be determined if the relative cost of false positives and false negatives is known. Here, we choose three representative scenarios. Each scenario has a requirement and error rate (defined in Main Text Table 2). We identify the threshold, $\hat{p}$, at which the requirement is most closely exceeded (i.e. if the

requirement were, hypothetically, that an error rate should be a maximum 15%, the threshold that produces an error rate below 15% but as close to 15% as possible will be chosen).

In Scenario 1, we do not consider epidemiological context but simply minimise false negative and false positive rates equally. We do this by maximising the two correct classification rates both individually and in total, as measured by the harmonic mean. The harmonic mean is used widely in the classification literature as it is maximised by achieving large values in all its component parts, rather than the arithmetic mean which can be maximised by having one extremely large component at the expense of other components. In other words, the arithmetic mean could be large because it has a very high TPR but a small TNR, whereas the harmonic mean will maximise both TPR and TNR. While conceptually the harmonic mean is better suited than the arithmetic for this use case, both produce qualitatively the same results for these data.

Scenario 2 corresponds to the situation in Bangladesh at time of writing (September 2021), with COVID-19 cases beginning to rapidly increase again. Under these circumstances, false negatives are extremely costly relative to false positives due to the exponential growth of the disease.

In Scenario 3, the pandemic is not declining but maintaining a steady rate of cases. In this situation, policy-makers may be keen to keep false positive diagnoses low to prevent lockdown fatigue and to keep the workforce active.

The requirements in Scenario 2 and 3 were developed in discussion with the Institute of Epidemiology, Disease Control and Research (IEDCR), Bangladesh, for illustrative purposes.

3.2. Data Collection

This document compiles the Community Support Teams' Standard Operating Procedures for the identification of potential COVID-19 patients, screenshots of the data-collection application, and the protocol for the taking of nasal swabs for rapid antigen and PCR testing.

Standard Operating Procedure (SOP) 1 c_for support for Community Support Teams to identify the vulnerable/Risk group

204

Preamble:

The Community Support Teams (CST) must be able to follow all the protocols involved in Phase-II of community surveillance of COVID19, in order to control the spread of Coronavirus and find out and protect the vulnerable individuals.

Important Definitions:

Potential Virus Fighter (PVF): An individual reporting symptom of COVID-19. We will be identifying PVFs in the following ways:

a) through visiting household everyday looking for PVF

b) through individuals calling government hotlines 333) and reporting any symptom;

c) other household members of the PVF/VVF who showed symptom being screened

Once the PVF has been screened, the result can be either of these three:

Verified Virus Fighter (VVF): A PVF who has been screened and has high fever and with relevant signs/symptoms of respiratory disease (for example cough, shortness of breath (in last 15 days), sore throat) or the loss of the sense of smell.

OR an individual who tested COVID-19 positive in the last few days.

PVF with follow-up: A PVF who has been screened and whose body temperature is between 99.0oF to 99.4oF AND who has at least one sign/symptom of respiratory disease (for example cough, shortness of breath (in last 15 days), sore throat, the loss of the sense of smell).

Cleared Virus Fighters (CVFs): A PVF whose body temperature is below 99oF or who does not exhibit any symptoms of respiratory disease (for example cough, shortness of breath, sore throat, the loss of the sense of smell).

Vulnerable Individuals:

Certain individuals are at higher risk of developing complications and dying from COVID-19, these include older individuals (50 years or older in the context of Bangladesh), diabetics, hypertensive individuals, individuals with respiratory diseases such as COPD or those with compromised immune systems. Pregnant women are also a high-risk group for COVID-19 related adverse outcomes.

Scope

For use by CSTs, AMS/VAMs, telemedicine doctors, field Implementation teams and their support teams operating in urban & rural areas of Bangladesh to carry out surveillance for COVID19 and identification and protection of vulnerable groups.

Purpose

The purpose of this Standard Operating Procedure (SOP) is to provide a brief overview of the workflow of CSTs work to a) identify vulnerable individuals efficiently, b) identify the PVF c) take the necessary steps to follow once someone identified as VVF, CVF or follow up PVF. The SOP will link to other technical SOPs and provide guidance to the following activities.

Specific Objectives

1. Reach out to the community and attempt to make contact with households
2. Screen all individuals with COVID-like symptoms (PVFs) to identify if they are VVFs
3. Provide counselling on home management of COVID-19, ensuring 14-day quarantine, mask-wearing guidance to VVFs, and also refer them to CST telemedicine or hospital depending on the severity.
4. Screen all individuals 35 years and older and any pregnant women to identify vulnerable individuals (over 50 years, diabetics, hypertensives, individuals with COPD and pregnant women)
5. Provide counselling on specific protection measures for vulnerable individuals and referral to telemedicine for management of morbidities (diabetics, hypertensive, etc) and pregnancy.

6. Provide SRHR telemedicine numbers to all females aged 15-49 years in the household.

Procedure:

Once the[206] CSTs are trained and grouped into teams and assigned to a particular ward, they will need to do the following coordination activities:

A. Coordination with local authorities Urban:

The AM/VAM (with support from the Field Implementation (FI) team of BRAC will organize in-person meetings for the different wards and zones of the city corporation. The participants should include: the focal person from the ward councilor, the ward councilor, and the zonal executive officer (ZEOs) and the Deputy Chief Health Officer (DCHO). The AM/VAM will support the FI team to inform the local police station about the CSTs working under their jurisdiction. This will include sharing a list of each CST member (along with their photos) working in their particular wards.

Steps in organizing the meeting:

a) They will be provided the contact details of focal persons and members from Ward councillors.

b) All physical distancing rules have to be followed: the meeting will only include essential individuals to prevent overcrowding. There should be a minimum of 1-metre distance between each individual.

c) All participants will perform hand hygiene on arrival and when leaving the meeting and they will all wear masks

d) Prior to the commencement of the meeting, the meeting venue, including chairs and tables, will be cleaned with disinfectants, especially hard surfaces.

e) Keep the meeting as brief as possible, try to finish within 20-30 minutes

f) CST members will check each participant's body temperature before the meeting and maintain hand hygiene throughout the meeting. Participants with whose body temperature is over 99.0oF should be screened as a PVF and cannot join the meeting

Follow-up: On a regular basis, the CST should share activity updates with the local authority /focal person through telephone. The AMs/VAMs will update the FI team regularly, who will also facilitate discussion with the ward councilors/ZEOs/DCHO.

207

Follow-up: On a regular basis, share activity updates with the local authority through telephone.

Note: Representatives from the different partner organizations will try to attend the field coordination meetings.

B. Maintenance/Handling of logistics

Refer to SOPS 3 and 5 for materials needed for CSTs to carry out their duties in a safe and professional manner.

CST members will be provided with an Infrared thermometer, oximeter, Wrist watch BP monitor, three-layer cloth mask, measuring tape, gloves, goggles, bleaching powder, disinfectant containing 70% alcohol, id card and vest. They can keep the logistics in house of one of the members. Infrared thermometer, oximeter, three-layer cloth mask will be provided in the training by FAO. Other logistics like more masks and sanitizers will be provided and managed by BRAC.

B. **Contacting PVFs**

1. **Word-of-mouth:**

The CSTs will aim to visit an agreed number of households (but the focus should be on complete and comprehensive screening - it is more important to identify VVFs and vulnerable than to maximize household visit numbers). During the visit they will identify PVFs by word of mouth.

2. **Government Hotline:**

- The CSTs will also have to visit households with PVFs identified through the government hotline. The AM/VAM will contact the PVFs who called in Government hotlines in the last 2-3 days using the phone number used in the call. They will communicate with individuals, guided by a talking point tree, which explains the CST activities and requests the person to allow a screening visit from the CSTs. The contact information will be passed to the specific CST team through the CST mobile app.
- The CST will receive a list of phone numbers of PVFs in their CST mobile app.

- One of the CST members will call the PVF, introduce themselves, describe the purpose of visiting their house and request to schedule a visit. (They will follow the leaflet on FAQ "Coronavirus and CST team related information).

208

- If the PVF is reluctant for a visit, the CST member will try to convince them using interpersonal communication skills. If they still do not agree for the household visit, then the CST member will try to advise them about quarantine over the phone and also ask if they require food or medicine support. The CST will also check if there is any pregnant woman in the house, or any woman who has given birth in the last 6 weeks.

Note: The CST (AM/VAM) should make every effort to contact the PVF, this includes calling each number three times before giving up if it is not answered. If the individual is not willing to have a home visit, the CST will try to counsel them; this may include two calls to try to arrange a home visit.

D. The total Household Visit will include three major activities:

1. Household Form 1

2. PVF Screening (Please follow the SoP 3 _Quarantine Screening 31 August'20 V8.docx and SoP 5_Home Family Quarantine support_310820 V8.docx)

3. Vulnerable Screening

Household Form

1. After arriving at a house, the CST will ask to speak to an adult household member and note down the household address in the CST app.

2. The CST will explain about the CST programme, about COVID-19 precautions, and provide CST related leaflet and stickers

3. At this point, the CST will seek consent if the household agrees to a health screening and their information to be passed to health services.

4. Consent Statement: Take consent from the responder. If the household agrees to a health screening and their information to be passed to health services.

5. If the household member doesn't agree then the CST will continue to finalize the form without collecting any phone number and name, give them the CST sticker, thank them and leave the house.

209

6. If the household member agrees to continue, the CST will note down then his/her name and phone number as the primary respondent and proceed with the rest of the household form questions (basic questions about vulnerability: Age breakdown of HH members (most important to obtain accurate information about number of individuals over 50 years of age); pregnant, hypertension, diabetes and COPD).

PVF Screening:

7. The CST will then screen each member with COVID-19 symptoms with the PVF screening form will need to be completed for each PVF.

8. At first, the CST will seek consent for PVF screening, if the person doesn't agree then the CST will end the PVF screening. If the person agrees, then the CST will continue with the PVF screening form. This PVF screening process will need to be repeated for each household member with COVID-19 symptoms.

Consent Statement:*"We will ask simple questions about your health and measure your temperature using an infra-red thermometer. All of your data will be kept confidential under the Ministry of Health and Family Welfare of Bangladesh. Your data might be shared for telemedicine referral and other health-related research or services.You have the right to stop this interview at any point in time or refuse to give answers to any questions that make you uncomfortable.."*

210

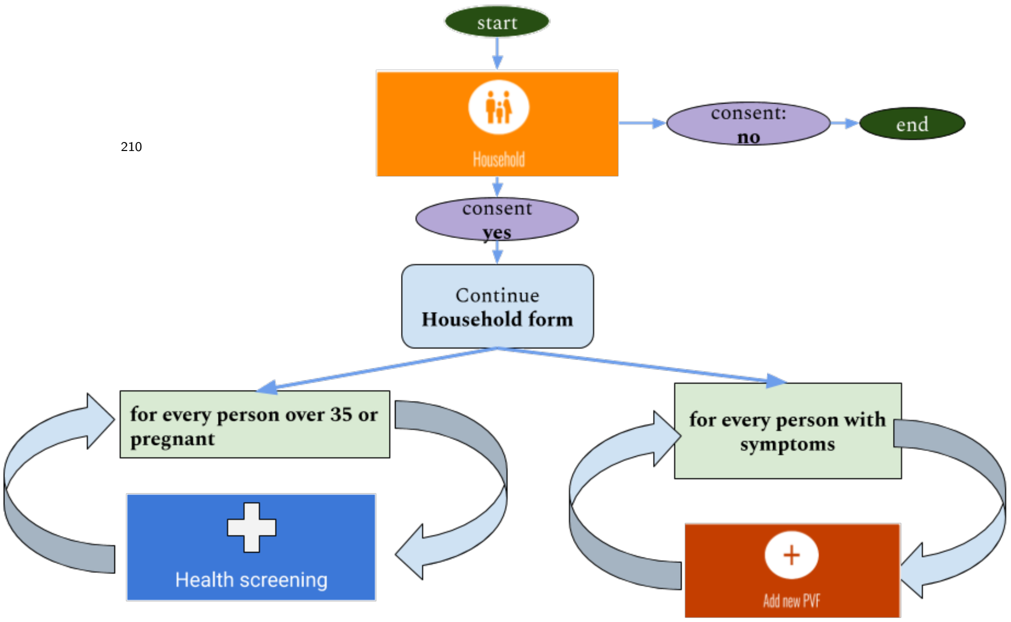

Vulnerable Screening

1. If there is anyone over 35 years of age or if there is a pregnant woman, then please offer them health screening. Please start with the oldest household member.
2. The CST will explain why it is necessary to identify vulnerable people.
3. The CST will follow the App to fill up the first part of the health screening form.
4. Depending on whether the person has one or more declared health issues, the CST will seek consent to do a physical examination to help identify undiagnosed conditions (for example, if an individual says no or does not know about having high blood pressure, the CST will measure their blood pressure).

Consent Statement:*Do you want to have a physical examination now, this will include you measuring your own waist and might include measuring blood pressure.*

5. Once the person agreed to the screening process, please follow SOP 4 c_on how to measure BP and SOP 4 d_on how to measure waist circumference.

6. The CST will proceed to ask remaining questions on the screening form.
7. At the end of the screening form, the CST should ask for the respondent's name and phone number.

C. Steps to take for individuals identified as vulnerable:

For individual identified as Elderly, Diabetic, Hypertensive and COPD- provide counselling based on SOP 09 and refer them to CST telemedicine.

Pregnant women: Refer to SRHR telemedicine

D. Steps to take after PVF screening based on the screening result

1. Steps to be taken if PVF is identified as VVF:

- Please refer to SOP 5 Home Family Quarantine Support". Support the VVF in maintaining quarantine for 14-days along with their entire households.
- Measure oxygen saturation levels and enter the level into the App. Take appropriate action based on the oxygen saturation levels.
- Connecting to the telemedicine doctor who will determine the severity of the VVF's symptoms. Depending on the severity, the doctor will recommend a course of action. (Refer to SOP 7: Dedicated Medical Guidance Call Centre for VVF). If it is not possible to connect to the doctor, the CST can leave the number with the VVF to call later.When connecting the VVF with a Telemedicine doctor CST needs to inform the doctor about this. Doctor will decide the severity of the case.
- Provide essential medicine and food support for low income households or arrange for these things to be procured by friends or neighbors.
    a. The CST will teach the other members of the household on how to avoid direct contact with VVFs while still supporting and motivating the VVF fight against COVID-19. The CST will ensure that the neighbors will understand the role of the VVF in the fight against COVID 19 and are ready to help them.

b. The CST will proceed to include information on all household members as per the app specifications (name, phone number, age, gender and relationship with the VVF). If another household member is showing COVID-19 symptoms and wants
212 to be screened as a PVF, only then the CST will screen them as a PVF, otherwise only the information mentioned above needs to be collected for each household member.

c. Carry out scheduled follow up visits to ensure adherence to proper quarantine, check if medicines or food is needed and to check if symptoms have worsened.

- At the 14th day of quarantine period, doctors from telemedicine will call VVF to find out the current status if no further sickness is in the household, they are all free to end isolation.

2. Steps to be taken if the PVF is identified as PVF with follow up

The CST will counsel them about monitoring symptoms very closely, and call the CST right away if l if the symptoms worsen

a. The CST will share their phone numbers if they need further support and will advise of any follow up visits

b. In any event, the CSTs will revisit him/her within two days to reassess their symptoms and start the whole screening processes again by following the relevant section in the App.

c. The App will determine if the person is VVF, PVF with follow up or cleared PVF;

d. If the app changes the status of the PVF with Follow-up to VVF, then the CST will follow SOP 5 as outlined above.

e. If the app keeps the status of the PVF with follow up, the CST will ask the PVF to contact them if the symptoms worsen. If PVF doesn't contact the CST, CST does not need to visit the household further.

f. Cleared PVF, the CST will follow the SOP 5 for these categories.

g. The CST will advise the PVF with follow-up to call the CST immediately if symptoms worsen. The CST will always also advise the entire household to wear masks when going outside their homes and to request visitors to wear masks when visiting.

h. If anyone in the family develops cough or fever, they may report again contacting their local CST or using 333 or 16263.

3. Monitoring VVFs and PVFs with follow-up:

- Monitoring visits will clearly schedule and are designed to:

  a. check the health status of the household

  b. check for compliance with isolation; this should include problem solving if the family are having trouble access food or medicines

  c. Ensure that the family are not being subjected to stigmatization from neighbours.

Standard Operating Procedure (SOP) 3 for Screening Potential Virus Fighters

Preamble

Once a PVF[214] is identified and details entered into the CST Mobile App, the job of the CST is to screen the CST for COVID19 as soon as possible. The screening process is assisted by the App, which will confirm the status of the PVF.

Important Definitions:

Potential Virus Fighter (PVF): An individual reporting symptom of COVID-19. We will be identifying PVFs in the following ways:

a) through word of mouth from the community

b) through individuals calling government hotlines 333) and reporting any symptom;

c) other household members of the PVF being screened

Once the PVF has been screened, the result can be either of these three:

1. **Verified Virus Fighter (VVF):** A PVF who has been screened and has high *fever* and with relevant signs/symptoms of respiratory disease (for example cough, shortness of breath (in last 15 days), sore throat) or the loss of the sense of smell.

   OR an individual who tested COVID-19 positive in the last few days.

2. **PVF with follow-up:** A PVF who has been screened and whose body temperature is between 99.0^{o}F to 99.4^{o}F AND who has at least one sign/symptom of respiratory disease (for example cough, shortness of breath (in last 15 days), sore throat, the loss of the sense of smell).

3. **Cleared Virus Fighters (CVFs):** A PVF whose body temperature is below 99°F or who does not exhibit any symptoms of respiratory disease (for example cough, shortness of breath, sore throat, the loss of the sense of smell).

Vulnerable Individuals:

Certain individuals are at higher risk of developing complications and dying from COVID-19, these include older individuals (50 years or older in the context of Bangladesh), diabetics, hypertensive individuals, individuals with respiratory diseases such as COPD or those with compromised immune systems. Pregnant women are also a high-risk group for COVID-19 related adverse outcomes.

Community Support Team (CST): In urban and residential areas, the CST will consist of at least two volunteers from different volunteer organizations (e.g., Platform, CDP, Utshorgo foundation, Young Bangla[215]), students from the communities and/or volunteers nominated by the Ward councilors.

In urban slum area the CST will consists of two Shasthyo Kormi (SK) from BRAC.

Each CST team will be assigned to one ward, and they will be supervised by Area Managers (AM) or Volunteer Area Managers (VAM).

These AMs/VAMs will be responsible for multiple wards (and hence multiple CSTs).

Scope

The SOP is used to determine the status of PVFs by identifying signs and symptoms of COVID19. The PVFs may be identified by the hotline, the community, or as close contacts of a VVF or identified COVID19 patients.

Purpose

The purpose of this SOP is to provide detailed guidelines to the CST on how to screen **PVFs**) to determine if they are

a) VVFs

b) PVF with follow-up

c) Cleared PVF

Logistics required (in necessary quantity as per visit plan) for CST:

- Smart phone/tab
- Soap and clean water or alcohol-based hand rub
- PPE items (mask, goggles, gloves)
- Infrared thermometer
- Oximeter
- Biohazard bag/thick poly bag/covered container
- A bucket of prepared diluted 0.2% sodium hypochlorite/bleach solution
- All materials required to make a diluted 0.2% bleach solution for demonstration if PVF is found to be a VVF

- All necessary supportive medicines (first line treatment advised by government telemedicine number 16263) will be carried for distribution to VVF household.
- Phone numbers of local ME

216

Procedure

1. The CSTs will make a daily plan for household (Khana) visits to identify PVF by word of mouth as part of their daily work and the PVFs on the App provided by Area managers.
2. The CST will visit household as per given daily target and look for PVFs to be screened.
    - CST will start the conversation with permission and introduce themselves, explain the HH members why they are here.
    - Then they will ask if someone in the household is sick or want to be screened as a PVF
    - If there is any sick person or the member of household want to be screened, please follow the steps from “6 to 14”
    - If the household denied for screening please follow steps “4 and 5”.
3. The CST calls the number from the app to confirm the name and number of the PVF and to request a screening visit. If the number does not answer, they should try three times before reporting the number as not answering.
4. If the PVF is reluctant for a visit, the CST member will try to convince them using good interpersonal communication skills. If they still do not agree for the household visit/screening, then the CST member will try to advise them about quarantine over the phone and also ask if they require food or medicine support. The CST will also check if there is any pregnant woman in the house, or any woman who has given birth in the last 6 weeks. The CST should not give up on the home visit but call again at another time.
5. The CST should use good communication skills to build trust with the PVF and household members before starting the screening. They should remember to treat the PVF as an equal and to respect his/her concerns. They should explain clearly why they are wearing PPE and why they will be taking measurements and asking questions. They cannot enter the house and commence the screening without permission of the household members.

6. Before entering the house, the CST members should wash their hands using soap and water or an alcohol-based hand rub prior to donning appropriate Personal Protective Equipment (PPE):
    a. Eye Protection: Goggles
    b. A clean three layer cloth mask
    c. A pair of new gloves
7. PVF should collect information in a respectful way and record the data on the CST mobile app. If the app does not work for some reason they will record the data in the given PVF interview form. The order of questioning and data collection will be guided by the app or interview form.

8. All personal data and a complete history of the PVF's symptoms should be recorded on the CST mobile app or the form.

9. The PVF's temperature should be taken with an infrared thermometer and the measurement recorded on the CST mobile app. CST members will point the thermometer in 3 centimeters distance from the forehead of the VVF. Please refer to SOP 4a Using the Infrared thermometer.

10. Based on the signs and symptoms and temperature reading, the mobile app will determine if the PVF meets the definition for a **VVF** or **PVF with follow-up** or a **Cleared PVF.**

11. The CST will also check if there is any pregnant woman, any woman who has given birth in the last 6 weeks (42 days) or any vulnerable people in the household.

12. **If the PVF is a VVF, then the CST will take the following steps:**

    a) The CST will measure the blood oxygen saturation of the VVF using the pulse oximeter and record the reading in the CST mobile app (see SOP 4b. Using the oximeter).

b) If the oxygen saturation level is equal to or below 93%, the CST should explain to the VVF that he needs specialized medical treatment and immediately call the AM/VAM
218 for assistance to take the VVF to hospital.

c) The CST should proceed to ask the rest of the questions as prompted in the CST mobile app.

c) The CST should add information (name, age, sex, telephone number and relationship to the VVF) of each household member in the CST mobile app.

d) These household members should be screened as PVFS.

e) The CST will check if any member of the household (including the PVF who was just screened) is either pregnant or a breastfeeding. The household will be given the OGSB number to call for any advice on referral to a hospital or any other issue. If a female household member is pregnant: The CST will advise them to go to a health facility for regular antenatal visit, and to deliver in their facilities. -If a female household member is a breastfeeding mother: The CST will advise them to wear masks while breastfeeding, and for them to consider family planning.

f) The CST should connect to the telemedicine doctor and hold the conversation on speaker phone so that both the VVF and CST can hear. The medical expert who will determine the severity of the VVF's symptoms and depending on the severity, will recommend a course of action. (Refer to SOP 9: Dedicated Medical Guidance Call Centre for VVF). If it is not possible to connect to the doctor, the CST can leave the number with the VVF to call later.

g) The CST should then follow the Home Family Quarantine Support SOP 5 for guidance on counselling the VVF and their family for maintaining 14-day home quarantine, implementing IPC within the household and support measures.

h) The VVF should be advised that there will be personal follow up visits on days 3 and 7 and then a phone check up on day 10.

219

i) The CST will then ask the VVF if they want to be tested for COVID-19, if the opportunity arises. Before recording the answer in the CST mobile app, the CST will clearly describe the consequences of agreeing to be tested as described below:

Please tell them that if they opt for testing, then the government (IEDCR) may call them for sample collection. If the family agrees to be contacted by the government for testing, then they might get a call from the government, and then two or more individuals might visit their house to collect the sample(s). These individuals might be wearing coveralls or other PPE items that might scare the neighbors. There is also a chance that the neighbors might ostracize/avoid the family because of this. If the VVF is less than 18 years old, then please ensure that their at least one of their parents/guardians is part of this conversation. The VVF and their family have complete autonomy to decide if they want to get tested or not. Their decision to test or not to test will not impact the services or support that they are supposed to receive from the CSTs.

Please refer to the Sample Collection SOP for details.

13. If the PVF is a <u>PVF with follow-up</u>, then the CST will take the following steps:

a) Counsel them on monitoring their symptoms and the importance of contacting the CST right away if symptoms worsen. They should advise on wearing masks outside the house for all household members
b) The CST will revisit the PVF with follow-up in two-days' time for re-screening.

14. If the PVF is a Cleared PVF, then the CST will take the following steps:

a) Counsel them on monitoring their symptoms and the importance of contacting the CST right away if symptoms worsen. They should advise on wearing masks outside the house for all household members.

b) The CST won't revisit the Cleared PVF again unless they call back for further support.

2. At this point, if there is any household member who is also reporting COVID-19 like symptoms and wants to be screened as a PVF, then the CST can screen this person as a PVF (recording this person as a new PVF screening in the CST mobile-app).

3. At the end of each household screening the CST members should remove the gloves carefully so as not to allow the outer surface of the gloves to contact their skin and then dispose of them appropriately in a covered container.
4. They should wash their hands using soap or clean with an alcohol-based hand rub before donning a new set of gloves.
5. Masks should be worn all day replaced whenever they become wet or visibly soiled and disposed appropriately in a thick poly bag/covered container. Refer to SOP 8 for correct donning and doffing of masks and mask care.

Standard Operating Procedure No 5 for Home Family Quarantine/Isolation of Verified Virus

Preamble

221

The isolation of the Verified Virus Fighter (VVF) and family is to prevent uncontrolled spreading of the virus responsible for COVID19. To ensure that the VVF and family are able to follow the 14 days isolation, he/she should be treated in such a way that he/she is comfortable and willing to remain in isolation. The other members of the household should be protected from COVID19 infection from the VVF through prevention control practices.

Important Definitions:

Potential Virus Fighter (PVF): An individual reporting symptoms of COVID-19. We will be identifying PVFs in the following ways:

a) through word of mouth from the community

b) through individuals calling government hotlines 333) and reporting any symptom;

c) other household members of the PVF being screened

Once the PVF has been screened, the result can be either of these three:

1. **Verified Virus Fighter (VVF):** A PVF who has been screened and has high *fever* and with relevant signs/symptoms of respiratory disease (for example cough, shortness of breath (in last 15 days), sore throat) or the loss of the sense of smell.

OR an individual who tested COVID-19 positive in the last few days.

2. **PVF with follow-up:** A PVF who has been screened and whose body temperature is between 99.0^{o}F to 99.4^{o}F AND who has at least one sign/symptom of respiratory disease (for example cough, shortness of breath (in last 15 days), sore throat, the loss of the sense of smell).

3. **Cleared Virus Fighters (CVFs):** A PVF whose body temperature is below 99°F or who does not exhibit any symptoms of respiratory disease (for example cough, shortness of breath, sore throat, the loss of the sense of smell).

Vulnerable Individuals:

Certain individuals are at higher risk of developing complications and dying from COVID-19, these include older individuals (50 years or older in the context of Bangladesh), diabetics, hypertensive individuals, individuals with respiratory diseases such as COPD or those with compromised[222] immune systems. Pregnant women are also a high-risk group for COVID-19 related adverse outcomes.

Scope

For use by Community Support Teams (CSTs) once a PVF has been declared a , VVF and recommended to follow 14 days of isolation.

Purpose

The purpose of this SOP is to provide guidance for CST on how to advise and support VVFs on self-isolation and on quarantine of household contacts.

The steps in screening of the PVF is covered in the SOP 3 Quarantine Screening.

Procedure

1. Once the **PVF** has been confirmed to be a **VVF** by the CST using the Mobile App, the CST should advise the VVF and family of his/her status and explain the role of the VVF in controlling the spread of COVID19. It is crucial that the CST explain the importance of his/her actions for the community and Bangladesh and gain the agreement of the VVFs and their families. Good communication skills are needed. Key points to be made include:

- The COVID19 virus is very contagious and can be spread through sneezing and coughing and touching contaminated surfaces. But the virus can easily be killed by cleaning and disinfection.
- Most people do not get very sick, but a small group may need to go to hospital.
- By isolating the VVF is preventing spread of the virus and is working to protect his/her community. If the virus spreads uncontrollably, the hospitals will be unable to cope and many people will die (can use the fish pond example).
- Isolation is a selfless act that helps others; the VVF is a hero.
- After 14 days of isolation the VVF should be over the COVID virus; it will also be clear if the other family members have also caught COVID19.
- The CST will support the family through the isolation period.

2. Discuss openly with the VVF any concerns and fears that he/she may have. Reinforce that the CST and ME are going to assist them. Explain clearly the assistance that the VVF can expect: this includes support visits, telemedicine, food bank support, access to basic [223] medicines and hospital transfer if needed,
3. VVF should be advised to go immediately into Family Quarantine/Isolate in their home with all household members (persons who live in the same home).
4. The process of isolation and quarantine should be carefully explained to the VVF and family in a supportive and non-threatening manner. Home Family Quarantine/Isolation means the VVF and their entire household have to adhere to the following conditions for the next 14-days:
   a. They should not leave their home for any reason.
   b. If, by chance, they do come across other people, they should stay at least 1 meter (3 feet) apart.
   c. They should not go out to buy food or collect medicine: they can ask the CST to support them in the process, ask someone else to drop off medicine or groceries at their home or order them by phone or online.
   d. They should not allow any visitors, other than the CST or medical persons, in their home.

Household hygiene

1. It is very important to protect other household members from COVID19. The VVF should strictly adhere to the following to prevent infecting other household members:
2. The VVF should remain isolated in a separate room and stay 1 meter (3 feet) from other members of the house.
3. He/she and must wear mask and also all family members must wear mask when more than one person is in a room.
4. Enough food and drinks should be prepared and delivered to the VVF's room but not handed to Him/her. Empty plates and cups should be placed into a bucket at the end of each meal and removed and washed in hot soapy water.
5. If the family uses common bathroom, specific bathroom times should be scheduled for the VVF and space given for him/her to move to the bathroom and back to the bedroom.

6. The family members should continue to communicate with the VVF and provide them with company and reassurance from a distance of 3 feet while wearing masks.

7. The VVF should not share dishes, drinking glasses, cups, eating utensils, bed linen, clothes or towels[224] with the rest of the family.

8. VVFs who are breastfeeding mothers can breastfeed their infants wearing a mask. They should thoroughly wash their hands with soap and water or sanitize their hands with alcohol-based hand rub before breastfeeding.

9. To reduce the spread of infection in the home, the VVF and other household members should do the following:
    a. Wash their hands with soap and water often, for at least 20 seconds, or use an alcohol-based hand rub when soap and water is not available. The CST members will show them how to correctly wash their hands and show a sample of alcohol based hand rub.
    b. The VVF should wear a cloth mask that covers the nose and mouth when he/she must be around other people or animals, including pets. The mask is not necessary when the VVF is alone.
    c. All the household members must wear masks at all times inside the house (except when someone is completely alone).
    d. All the household members should sneeze and cough inside the mask; for sudden onset of coughing or sneezing when they are not wearing the mask, they should cover their mouth and nose with a tissue or sleeve (not hands), put used tissues in the covered waste bin immediately and wash hands afterwards. If the mask gets soiled by cough or becomes wet it should be changed.
    e. The responsibility of taking care of VVF should be given to the healthiest family member who is without any comorbidities such as diabetes, hypertension, cancer, heart disease, chronic respiratory disease.
    f. Surfaces that are touched often (like door handles, bathrooms, kettles, light switches, chair arms) should be cleaned regularly using household cleaning products and disinfected with 0.2% bleach.. Electronic items such as phones should be cleaned with alcohol.

g. If a caregiver or other person needs to clean and disinfect a sick person's bedroom or bathroom, they should wear a mask and disposable gloves prior to cleaning. They should wait as long as possible after the VVF has used the bathroom before coming in to clean or use the bathroom. The area should be cleaned first with soap and water followed by disinfection with 0.2% bleach. The disinfected area should remain wet for 5 minutes and then excess bleach cleaned up with a clean cloth.

h. Wash the cloth mask with warm water and detergent every day or soak in 0.2% dilute bleach (Add 2 teaspoons of bleaching powder to 1 liter of water) for at least 1 minute, rinse with water, and then let air dry in sunlight if possible.

i. The house should be cleaned with normal household products, such as water and detergent, followed by a 0.2% disinfectant bleach. CST members will demonstrate how to make a 0.2% bleach solution (see below) and give each family a 250 mg packet of powder bleach to clean the surrounding surfaces around the VVF and the bathroom after use.

j. Used tissues and disposable cleaning cloths should be placed in garbage/ polythene bags and then put into a second bag and tied securely. The bag should be stored for 3 days before putting it in the outside bin. Other household waste may be disposed of as normal.

k. Laundry should be washed in the usual way. Laundry that has been in contact with an ill person can be washed with other people's items but they should not be shaken as this may spread the virus in the air.

10. To stay well while at home, the VVF and any ill household members should:

a. drink plenty of water to stay hydrated,
b. The VVF and family members should take Vitamin C, Vitamin D and zinc,
c. take paracetamol to help ease symptoms like fever and malaise,
d. stay in touch with family and friends over the phone or on social media, to help avoid feeling low or lonely
e. try to keep busy; -try activities such as , reading, online learning and watching films
f. do light exercise, if he/she feels well enough

11. If the household includes a vulnerable individual (someone who is 60 years old or over, has a long-term condition, is pregnant or has a weakened immune system), the household should try to move him/her to another house for 14 days.

12. If the vulnerable person must stay in the home, the VVF and the vulnerable individual should try to keep away from each other as much as possible by:

a. Keeping 1 meters (3 feet) away from each other,
b. Should wear mask all time even at home,
c. avoiding using shared spaces, such as kitchens or bathrooms, at the same time ,
d. opening windows in shared spaces, if possible, for air circulation,
e. cleaning a shared bathroom each time it is used, for example by wiping the touched surfaces with a disinfectant,
f. using detergent and warm water when washing dishes and dry everything thoroughly,
g. not sharing a bed, if possible,
h. not sharing towels, including hand towels.

13. If the VVF or another ill household member needs medical help during Family Quarantine/Isolation, he/she should not go to a clinic, pharmacy or hospital. He/she should stay at home and call the community support team or contact the telemedicine doctor dedicated to VVFs . The CST will have the number for the local ME.

14. The CST team members should follow up physically with the VVF and his/her household members on the 3rd and 7th day. During the visit they will check VVF's temperature, oxygen saturation and confirm if the VVF and his family are maintaining quarantine (please see SOP 1 Process Flow_Urban Areas for details) Data on body temperature, oxygen saturation, adherence to home quarantine, will be recorded through the CST mobile app during the follow-up visits. The CSTs will also follow-up through telephone on the 10th day to make sure VVF and his family maintained home quarantine properly and also to enquire if they require further food support/medical attention.

15. During the follow-up visits, the CSTs should counsel the VVF and the family again on steps 2-11 (to reinstate the importance of maintaining quarantine).

16. The CST will ensure that the neighbors understand the fight and are ready to help them morally and mentally to boost up VVF and the family.

Preparation of disinfectant bleach solution

1. A 0.2% bleach solution should be made fresh every day by the CST. They will need the following equipment:

- 10[227] litre bucket with a lid
- 20% bleach powder
- I teaspoon
- 1 plastic or wooden stirrer
- Measure for 500 ml (ie a water bottle)
- Protective equipment such as Mask, gloves, goggles. Solution must be made in an open environment.

2. Before beginning the dilution, wear the PPE.
3. To make 1 liter of bleach solution, pour 1 liter of water into the bucket. Add two teaspoon of bleach powder and mix with the stirrer. Immediately put the lid on the bucket
4. Experience will show how much is needed for a day's work but do not make more than be safely carried in the bucket. The calculation is easy: for 2 liters of water add 4 teaspoons of bleach; for 5 liters of water use 10 teaspoons of bleach powder
5. At the end of the day, pour out any remaining solution

3.2.1. Data Collection Mobile Application

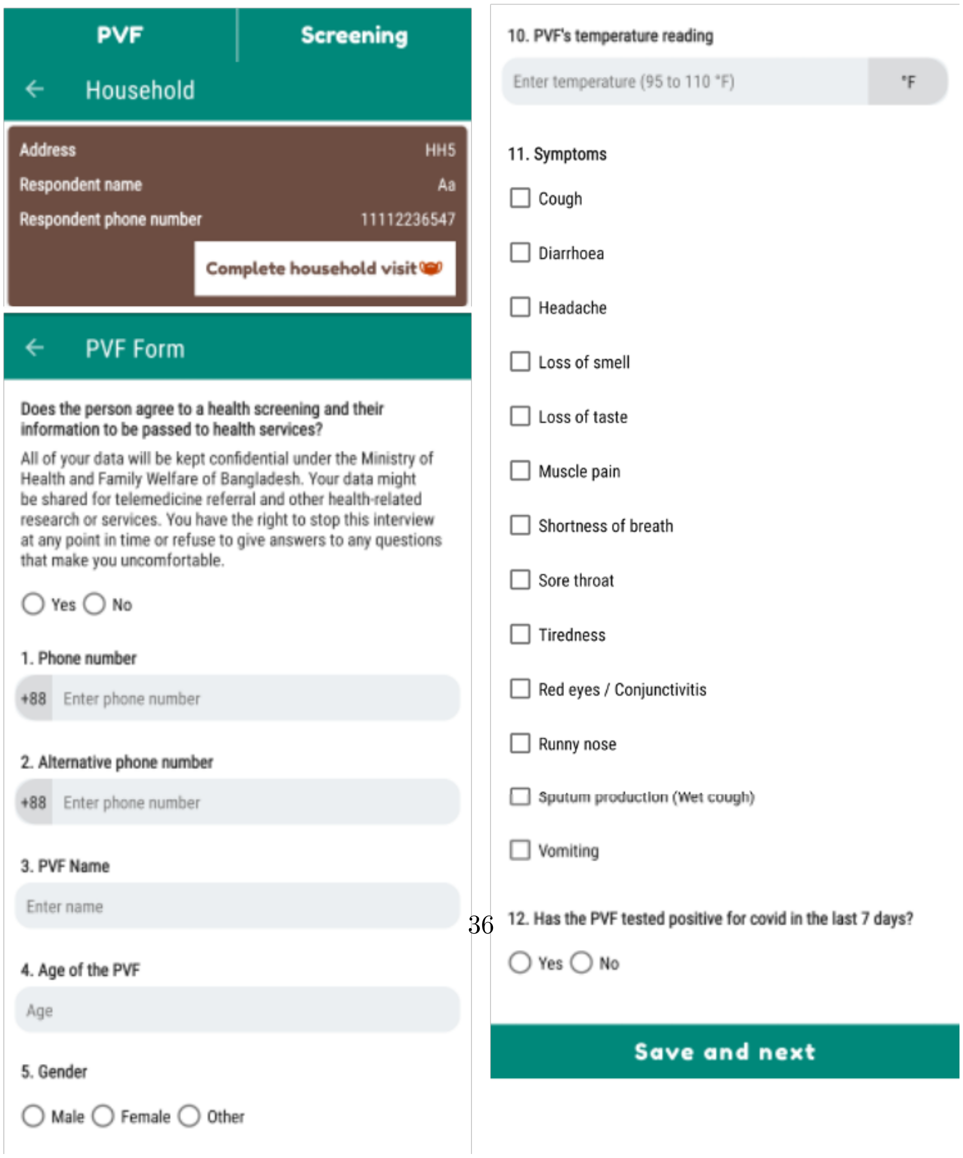

Prepared by Tasnuva Chowdhury
1/21/2021

Nasal Sample Collection and Testing Protocol

230

Questionnaire

**Name of Patient:**			
**Age:**	________ years or if <5 years ________ month	**Sex:**	☐ Male ☐ Female
**Occupation:**	Phone/Cell No	**Emergency Contact Number**	
**Address:**			
**Email Address:**		**NID**	
**Referred by:**		**Blood Group**	
**Payment status:**	☐ Free ☐ Paid		

COVID-19 Suspect Criteria

**Fever ≥ 38˚ C or 100.4˚ F**	Yes/No
**Headache**	Yes/No
**Cough**	Yes/No
**Breathlessness**	Yes/No
**Others (specify)**	

If any symptom present, date of first/earliest symptom onset: _____/_____/2020 (dd/mm/yy)

Clinical or radiological evidence of Pneumonia or Severe Acute Respiratory Distress Syndrome
Yes/No/Unknown

Has the person had contact with a confirmed case in the 14days prior to symptom onset?
Yes/No/Unknown

Has the person visited any health care facility in the 14 days prior to symptom onset?
Yes/No/Unknown

Concurrent risk factors (Check all that apply):
COPD/Asthma/Interstitial Lung Diseases/DM/IHD/HTN/CKD/CLD/Malignant diseases/ On steroid therapy/Pregnancy/Others _____________

Prepared by Tasnuva Chowdhury
1/21/2021

Nasal sample collection (infographics showing Left Nasal sample collection) instruction for CST 1

231

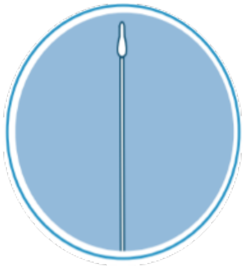

- ☐ Take out the nasal mid-turbinate swab from the packet and keep the tube safely for the time being.
- ☐ Touch only the plastic shaft not the padded end.

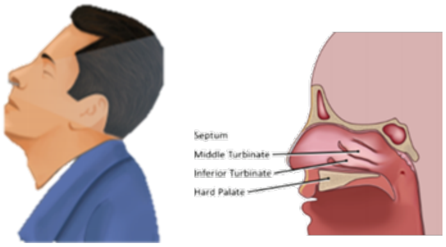

- ☐ Ask the patient to sit straight and tilt the head back (approximately 70 degree).

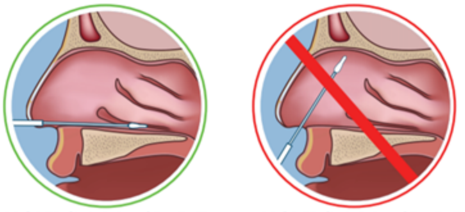

- ☐ Insert the swab in the nasal spare parallel to the hard palate.
- ☐ Resistance will be felt and that is the confirmation of reaching to the nasopharynx.

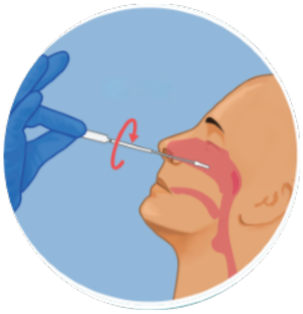

- ☐ Once the swab is against the hard surface rotate it several times.

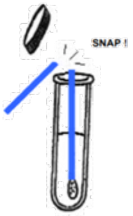

- ☐ Take out the swab from the left nose and insert the swab into the VTM labelled as "N"
- ☐ Make sure the liquid transport medium covers the tip of the swabs.
- ☐ Break the swab shafts at the marking on the shaft.

232

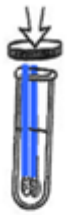

☐ Screw the caps back on the test tubes tightly.

☐ Once the nasal sample is collected by CST 1, CST 2 will check the box in the app (See example below).

Specimen:

☐ Collected ☐ Not collected

If collected mention type:

☐ Right nasal swab ☐ Throat swab ☐ Saliva Combined left nasal swab and throat swab

Nasal swab sample analysis

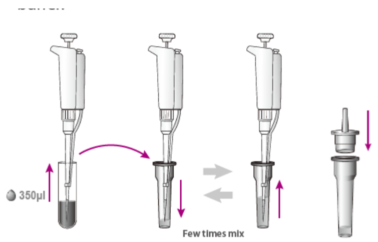

☐ Using a micropipette, collect the 350µl of specimen from the VTM. Mix the specimen with an extraction buffer in anther tube.

☐ Press the nozzle cap tightly onto the tube.

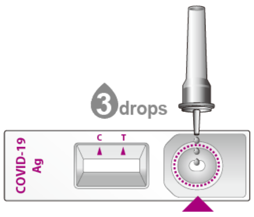

☐ Apply 3 drops of extracted specimen to the specimen well of the test device.

233

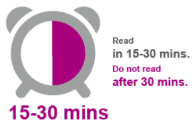

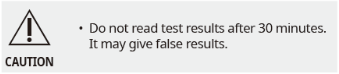

☐ Read the test result in 15-30 minutes.

Interpretation of Nasal sample analysis

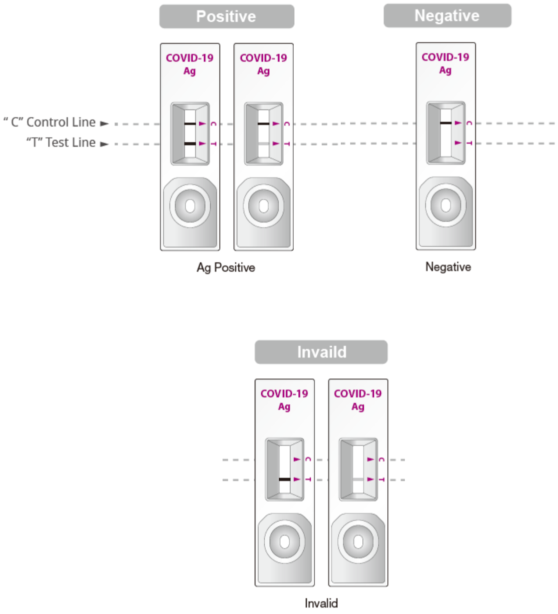

☐ A colored band, control line (C), in the top section of the result window will appear in positive and negative test result.

- o Presence of a second colored band, "T" test line, in conjunction with the "C" Control line is always considered as positive. Even if the "T" test line is faint.
- o Presence of only "C" control line with out "T" test line will be considered as negative.

☐ Absence of the control line in the top section will always consider the result as invalid.

Image Source: CDC. Reference to specific commercial products, manufacturers, companies, or trademarks does not constitute its endorsement or recommendation by the U.S. Government, Department of Health and Human Services, or Centers for Disease Control and Prevention

References

[1] James H Albert and Siddhartha Chib. Bayesian analysis of binary and polychotomous response data. *Journal of the American statistical Association*, 88(422):669–679, 1993.

[2] Bob Carpenter, Andrew Gelman, Matthew D Hoffman, Daniel Lee, Ben Goodrich, Michael Betancourt, Marcus Brubaker, Jiqiang Guo, Peter Li, and Allen Riddell. Stan: A probabilistic programming language. *Journal of statistical software*, 76(1):1–32, 2017.

[3] Tilmann Gneiting and Adrian E Raftery. Strictly proper scoring rules, prediction, and estimation. *Journal of the American statistical Association*, 102(477):359–378, 2007.

[4] Daniel Lewandowski, Dorota Kurowicka, and Harry Joe. Generating random correlation matrices based on vines and extended onion method. *Journal of multivariate analysis*, 100(9):1989–2001, 2009.
