# Supplementary material for: Combining rapid antigen testing and syndromic surveillance improves community-based COVID-19 detection in a low-income country
Source: Nat Commun. 2022 May 26;13:2877. doi: 10.1038/s41467-022-30640-w (PMC9135686; doi:10.1038/s41467-022-30640-w)
Supplement: Supplementary file 3 — Reporting Summary [file 41467_2022_30640_MOESM3_ESM.pdf]

nature portfolio

Corresponding author(s): Fergus J Chadwick

Last updated by author(s): Jan 6, 2022

Reporting Summary

Nature Portfolio wishes to improve the reproducibility of the work that we publish. This form provides structure for consistency and transparency in reporting. For further information on Nature Portfolio policies, see our Editorial Policies and the Editorial Policy Checklist.

Statistics

For all statistical analyses, confirm that the following items are present in the figure legend, table legend, main text, or Methods section.

n/a	Confirmed	
☐	☒	The exact sample size (*n*) for each experimental group/condition, given as a discrete number and unit of measurement
☐	☒	A statement on whether measurements were taken from distinct samples or whether the same sample was measured repeatedly
☐	☒	The statistical test(s) used AND whether they are one- or two-sided<br>*Only common tests should be described solely by name; describe more complex techniques in the Methods section.*
☐	☒	A description of all covariates tested
☐	☒	A description of any assumptions or corrections, such as tests of normality and adjustment for multiple comparisons
☐	☒	A full description of the statistical parameters including central tendency (e.g. means) or other basic estimates (e.g. regression coefficient) AND variation (e.g. standard deviation) or associated estimates of uncertainty (e.g. confidence intervals)
☒	☐	For null hypothesis testing, the test statistic (e.g. *F*, *t*, *r*) with confidence intervals, effect sizes, degrees of freedom and *P* value noted<br>*Give P values as exact values whenever suitable.*
☐	☒	For Bayesian analysis, information on the choice of priors and Markov chain Monte Carlo settings
☒	☐	For hierarchical and complex designs, identification of the appropriate level for tests and full reporting of outcomes
☐	☒	Estimates of effect sizes (e.g. Cohen's *d*, Pearson's *r*), indicating how they were calculated

Our web collection on statistics for biologists contains articles on many of the points above.

Software and code

Policy information about availability of computer code

Data collection	"CST Intevention Program" 2.0 application (https://play.google.com/store/apps/details?id=com.icddrb.cstlive)
Data analysis	R version 4.0.1, Stan 2.28.2, CmdStanR 0.5.1 all code is available at https://github.com/fergusjchadwick/COVID19_SyndromicRAT_public

For manuscripts utilizing custom algorithms or software that are central to the research but not yet described in published literature, software must be made available to editors and reviewers. We strongly encourage code deposition in a community repository (e.g. GitHub). See the Nature Portfolio guidelines for submitting code & software for further information.

Data

Policy information about availability of data

All manuscripts must include a data availability statement. This statement should provide the following information, where applicable:

- Accession codes, unique identifiers, or web links for publicly available datasets
- A description of any restrictions on data availability
- For clinical datasets or third party data, please ensure that the statement adheres to our policy

All publicly available data is accessible at https://doi.org/10.5281/zenodo.6422756

Field-specific reporting

Please select the one below that is the best fit for your research. If you are not sure, read the appropriate sections before making your selection.

☒ Life sciences ☐ Behavioural & social sciences ☐ Ecological, evolutionary & environmental sciences

For a reference copy of the document with all sections, see nature.com/documents/nr-reporting-summary-flat.pdf

Life sciences study design

All studies must disclose on these points even when the disclosure is negative.

Sample size	Sampling took place during a defined period (19th May - 11th July 2021). All qualifying cases (see data exclusions) during this period were included. These dates were selected based on the timing of ethical approvals and the timescale needed to train the community support teams (CST) to collect samples and accompanying epidemiological metadata as well as the testing capacity available which was affected by the rapid rise in COVID-19 cases during this period. The sample sizes were shown to be sufficient for the models used through temporally-structured cross-validation to assess predictive power..
Data exclusions	Individuals who did not meet the following criteria were excluded: 16 years or older, fever of >38°C, one or more of the target symptoms (breathing problems, cough, diarrhea, ongoing fever, headache, loss of smell, loss of taste, muscle pain, red eyes, runny nose, sore throat, tiredness, vomiting and wet cough)
Replication	Our study did not involve an experimental intervention. The key finding was model predictive performance which was replicated using temporal cross-validation to ensure that performance was maintained outwith model training data. Five cross-validation sets were used, corresponding to approximately 10 day time intervals. This process reflects the real-world prediction of predicting COVID-19 status using recent cases for new patients. We emphasise in the paper that specific parameter estimates (e.g. the most predictive symptoms) are specific to this focal population at this time and that while the methods do generalise to other populations, specific results are unlikely to.
Randomization	Covariate information (age, gender and symptom profile) was used in our models. As this was a prospective, diagnostic study there was no need for randomization as there was no treatment/group allocation.
Blinding	Blinding not needed as there was no group allocation within the study.

Reporting for specific materials, systems and methods

We require information from authors about some types of materials, experimental systems and methods used in many studies. Here, indicate whether each material, system or method listed is relevant to your study. If you are not sure if a list item applies to your research, read the appropriate section before selecting a response.

Materials & experimental systems

n/a	Involved in the study	
☒	☐	Antibodies
☒	☐	Eukaryotic cell lines
☒	☐	Palaeontology and archaeology
☒	☐	Animals and other organisms
☐	☒	Human research participants
☒	☐	Clinical data
☒	☐	Dual use research of concern

Methods

n/a	Involved in the study	
☒	☐	ChIP-seq
☒	☐	Flow cytometry
☒	☐	MRI-based neuroimaging

Human research participants

Policy information about studies involving human research participants

Population characteristics	Recruitment took place across low-income communities in Dhaka North Community Corporation between 19th May 2021 and 11th July 2021. Participants were identified for COVID-19 testing by community support teams (CSTs). Participants were between the ages of 16 and 95. 46% identified as female and 54% identified as male. No genotypic or comorbidity information was collected. All patients selected were symptomatic, 22% tested positive for COVID-19 using PCR. Full breakdown of population characteristics by age, gender and symptoms are presented in Table 1.
Recruitment	Community Support Teams (CSTs) deliberately target low-income communities. CSTs are community-based volunteer health workers trained to identify individuals reporting symptoms suggestive of COVID-19 through hotline calls or community-based reporting channels. These individuals and their neighbours are then assessed by the CSTs and interviewed to find suspected additional cases in the community. Probable cases identified by CSTs are counselled to isolate for 14-days under household quarantine, connected to telemedicine services for home-based COVID-19 management, and provided with over-the-counter medication or medical referrals if the case is severe. As a result, recruitment is biased towards patients who are willing to avail themselves of the CST services, i.e. more ill individuals. However, we emphasise in the manuscript that we are

	developing a methodology for predicting COVID-19 in a defined group and the methodology should be applied to new patient groups as needed (i.e. the models should be refit to other populations, the findings from this population in terms of parameter estimates or most predictive symptoms should not be assumed to translate).
Ethics oversight	The study protocol was approved by the Institutional Review Board at the IEDCR, Ministry of Health, Bangladesh, IEDCR/IRB/04.

Note that full information on the approval of the study protocol must also be provided in the manuscript.
